# Maternal dietary free or bound fructose diversely influence developmental programming of lipogenesis

**DOI:** 10.1186/s12944-017-0618-z

**Published:** 2017-12-01

**Authors:** Armagan Aytug Yuruk, Reyhan Nergiz-Unal

**Affiliations:** Department of Nutrition and Dietetics, Faculty of Health Sciences, Hacettepe University, 06100 Ankara, Turkey

**Keywords:** Fetal programming, Fructose, Insulin, Non-esterified fatty acids, Triglyceride

## Abstract

**Background:**

Maternal dietary choices throughout preconception, pregnancy, and lactation irreversibly affect the development of fetal tissues and organs, known as fetal programming. Recommendations tend to emphasize reducing added sugars. However, the impact of maternal dietary free or bound fructose in added sugars on developmental programming of lipogenesis is unknown.

**Methods:**

Virgin Sprague-Dawley rats were randomly divided into five groups. Rats were given feed and plain water (control) or water containing maltodextrin (vehicle), fructose, high-fructose corn syrup (HFCS) containing 55% fructose, sucrose (20% *w*/*v*) for 12 weeks before mating and throughout the pregnancy and lactation periods. Body weight, water, and feed intake were measured throughout the study. At the end of the lactation period, blood was drawn to determine the fasting levels of glucose, insulin, triglycerides, and non-esterified fatty acids (NEFA) in blood. Triglycerides and acetyl Co-A Carboxylase-1 (ACC1) levels in livers were analyzed, and insulin resistance was calculated.

**Results:**

The energy intake of dams in the HFCS group was higher than in the fructose group, while weight gain was less in the HFCS group than in the fructose group. HFCS resulted in greater insulin resistance in dams, whereas free fructose had a robust effect on the fetal programming of insulin resistance. Free fructose and HFCS in the maternal diet increased blood and liver triglycerides and NEFA content in pups. Furthermore, fructose and HFCS exposure increased phosphorylated ACC1 as compared to maltodextrin and control, indicating greater fatty acid synthesis in pups and dams.

**Conclusion:**

Different types of added sugar in the maternal diet have different metabolic effects on the developmental programming of lipogenesis. Consequently, high fructose intake via processed foods may increase the risk for chronic diseases, and free fructose might contribute to developmental programming of chronic diseases more than bound fructose.

## Background

High levels of intake of added sugar has been connected to many chronic diseases i.e. obesity [1], metabolic syndrome [2], type II diabetes mellitus [3], cardiovascular diseases [4] and fatty liver diseases [5]. Recent work indicates that adult chronic diseases might be correlated with prenatal and maternal nutrition [6–8]. Exposure to a maternal diet with high levels of added sugar may increase the risk of adult obesity and insulin resistance of pups [9].

Independent of the amount consumed, the type of added sugar (sucrose, syrups etc.) might have a significant impact on the metabolic outcome. High intake of simple sugars might increase de novo lipogenesis, influence triglyceride production, and decrease fatty acid oxidation [10]. Fructose, a main component of added sugar, contributes more to lipid biosynthesis as compared to glucose [11]. In fructose metabolism, fructose bypasses the step requiring phosphofructokinase and does not stimulate insulin secretion as much as glucose; thus, high amounts of fructose contribute to de novo lipogenesis [12]. In lipogenesis, fatty acid synthesis includes the rate-limiting step catalyzed by Acetyl-CoA carboxylase (ACC1), which is controlled by insulin [13, 14]. However, the mechanisms underlying types of dietary added sugar types and lipogenesis is still unknown.

Elevated consumption of HFCS and the increasing prevalence of type II diabetes mellitus (T2DM) and obesity led to speculation that the free fructose in HFCS may contribute more to chronic diseases than bound fructose found in sucrose [15–17]. Although there are similar amounts of fructose in HFCS-55 (HFCS consisting 55% fructose) and sucrose, there are still questions as to whether HFCS contributes more to the progression of metabolic abnormalities, such as T2DM or cardiovascular disease (CVD) [18, 19]. Additionally, added sugars do not include naturally occurring sugars, such as fructose in fruits and lactose in milk. A limited number of studies have indicated that HFCS may have more negative metabolic effects than natural fructose consumed in fruit and honey [20, 21]. This might be due to the natural antioxidants, vitamins, minerals, and fiber consumed with natural D-fructose in foods.

Added sugars and syrups are those that do not naturally occur in the food, but are added during processing or preparation [22]. The major sources of dietary added sugars are beverages, cakes, desserts, and candies (except sugar free food and drinks) [22]. Globally, the most commonly used added sugars are sucrose, glucose/fructose syrups (HFCS-55), and crystal dextrose [22]. Worldwide recommendations for added sugar intake are less than 5–10% of daily total energy [22–25] such as in Turkey [26].

Consequently, dietary recommendations tend to emphasize reducing added sugars. However, whether the different types of added sugar have a similar metabolic impact on the developmental programming of lipogenesis has not yet been studied. Thus, the aim of this study was to investigate the effect of chronic maternal consumption of bound versus free fructose in three different types of added sugars (fructose, sucrose, and HFCS-55) on lipogenesis-related markers such as feed intake; body weight; circulating levels of glucose, insulin, triglycerides, and non-esterified fatty acids (NEFA); liver triglycerides; liver ACC1; and insulin resistance/sensitivity in dams and their pups.

## Methods

### Animals, experimental design and dietary exposures

All animals received ethical and humane care within the provisions of the “National Ministry of Food, Agriculture, and Livestock Regulations on the Protection and Welfare of Animals Used for Experimental and Other Scientific Purposes” and Institutional Guidelines. Experiments were approved by the Animal Ethics Committee of Hacettepe University, Ankara, Turkey (IRB Number: 2012/57–04). Power analysis was performed to calculate the required number of rats (*n* = 7) per group to determine dietary effects of added sugar on lipogenesis. To stabilize the milk yield for pups after birth, 7 offsprings from each dam was enrolled to the study regardless of gender.

Pathogen-free, 3-week-old, virgin Sprague-Dawley female rats (*n* = 35) were obtained from the Hacettepe University Experimental Animals Research and Breeding Unit (Ankara, Turkey). To facilitate food and water intake and to promote minimal sedentary movement patterns, the rats were maintained individually in transparent polycarbonate cages at 20–22 °C (12 h light/dark cycle; 45% relative humidity) throughout the study. All rats had ad libitum access to standard laboratory rat chow (Nukleon Laboratory Systems Inc., Ankara, Turkey) and water throughout the washout and experimental periods.

**Fig. 1:**
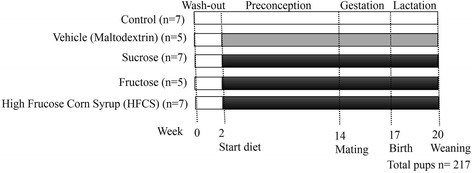
Schematic representation of the study design. Plain water (Control), and water with added maltodextrin (Vehicle: Maltodextrin), fructose (Fructose), high fructose corn syrup (HFCS), and sucrose (Sucrose). Values for “n” on the left represent the number of dams and “n” at the bottom represents the total number of offspring chosen

As summarized in Fig. 1, rats were divided into five groups after the washout period. The feed (by weight) consisted of 13% fat obtained from corn oil, 60% carbohydrates from corn starch and dextrose, and 24% protein (casein) with an energy content of 4.2 kcal/g (gross energy). The three groups were administered fructose (containing 100% free fructose), high fructose corn syrup (containing 55% free fructose; HFCS-55), or sucrose (containing 50% bound fructose) added to water for a final concentration of 0.2 g/mL (20% *w*/*v*) (0.8 kcal/mL). The control group had plain water, and the fourth group was the energy control (vehicle) and had an iso-caloric drink consisting of maltodextrin (0.2 g/mL (20% w/v)). The various combinations of water and added sugar were prepared fresh at the animal facility using autoclaved tap water. The energy and macronutrient composition of the diets per 100 g feed and 100 mL water is shown in Table 1. The energy content of the control diet was 415 kcal/100 g (only from feed), while the energy content for other four groups were 495 kcal/100 g (415 kcal from feed and 80 kcal from water). The administered protein and fat content were the same in all five groups (Table 1). The intake of feed and water was estimated by daily weighing of unconsumed feed and water. Body weights were measured three times per week.

**Table 1:** Energy and macronutrient composition of the diets administrated with feed and sweetened water during the dietary manipulation period

	Diet Groups				
Components	Control	Maltodextrin	Sucrose	Fructose	HFCS
Total Energy (kcal, %)	415	495	495	495	495
Energy, feed (kcal/100 g)	415	415	415	415	415
Energy, water (kcal/100 mL)	–	80	80	80	80
Total Carbohydrate (kcal, %)	249	329	329	329	329
Starch/dextrin, feed (kcal/100 g)	249	249	249	249	249
Added sugar, water (kcal/100 mL)	–	80	80	80	80
Total Carbohydrate (g, %)	62	82	82	82	82
Starch/dextrin, feed (g/100 g)	62	62	62	62	62
Added sugar, water (g/100 mL)	–	20	20	20	20
Protein feed (kcal/100 g)	112	112	112	112	112
Protein, feed (g/100 g)	28	28	28	28	28
Fat, feed (kcal/100 g)	54	54	54	54	54
Fat, feed (g/100 g)	6	6	6	6	6

HFCS: High Fructose Corn Syrup

After a 12-week dietary manipulation period, the 17-week-old, virgin female rats (dams) were paired with a Sprague-Dawley male rat and mating was confirmed by the appearance of a semen plug. All animals became pregnant; two rats failed to carry the pregnancy to the third week of gestation. The dietary manipulation continued in the same way during the pregnancy (3 weeks) and lactation (3 weeks) periods. Two rats were excluded from the study due to cannibalism during the lactation period.

### Collection of blood and tissues

Rats were fed until weaning (at the end of third week of birth) to determine the effects of maternal nutrition on pups. Also, pups were not fed with any type of feed (except breast milk) to eliminate any dietary effect other than maternal nutrition. At the end of the lactation period, 23-week-old dams and 3-week-old pups were deprived from feed and water for 5 h to obtain fasting blood profiles. They were anesthetized by subcutaneous injection of ketamine (0.1 mg/g body weight) and xylazine (0.02 mg/g body weight); blood and tissue samples (liver) were subsequently isolated and the animals were immediately euthanized by diaphragm puncture [27].

Blood was drawn from the vena cava into trisodium citrate (12.9 mM). The plasma samples were snap-frozen in liquid nitrogen and stored at −80 °C for further analysis. Livers were isolated from adherent tissue, rinsed with ice-cold saline to remove remaining blood, and snap-frozen in liquid nitrogen for further analysis [27].

### Measurement of glucose, insulin, free fatty acids and triglycerides

The levels of fasting glucose (Cayman Chemical Company, USA), insulin (Bertin Pharma, CNIM Company, Montigny-le-Bretonneux, France), NEFA (Cayman Chemical Company, USA), and triglycerides (Cayman Chemical Company, USA) were measured with enzymatic colorimetric commercial tests, following the manufacturers’ instructions by using an Absorbance Microplate Reader (ChromMate 4300, Awareness Technology Inc., USA). To measure the lipid content in the livers, liver lobes were homogenized with ice-cold saline by a microhomogenizator (T25 İka Labortechnik, Germany). Triglycerides in homogenized livers were measured by an enzymatic colorimetric method, normalized for protein content, and determined with the BioRad DC protein kit (BioRad Laboratories Inc., USA) [27].

### Estimation of insulin resistance/sensitivity

Homeostasis model assessment of insulin resistance (HOMA-IR = (fasting insulin (μU/mL) x fasting glucose (mmol/L)) / 22.5) and quantitative insulin sensitivity check index (QUICKI = 1/[log(fasting insulin (μU/mL) + log (fasting glucose (mg/dL))]) was calculated from fasting blood insulin and glucose levels [28–30].

### Measurement of ACC1 in the livers with western blotting

ACC1 and the phosphorylated form of ACC1 (p-ACC1) were measured by Western blot analysis of homogenized liver tissues. Liver tissues were lysed with ice-cold NP-40-based lysis buffer (pH 7.45) in the presence of protease and phosphatase inhibitors [31]. Protein in lysates was quantified with the BioRad DC protein kit (BioRad Laboratories Inc., USA). Samples with equal protein amounts were separated on 10% SDS-PAGE gels, and transferred to blotting membranes by semi-dry transfer. Membranes were immunoblotted with a primary anti-ACC1 rabbit mAb (1:1000) and secondary HRP-coupled goat anti-rabbit peroxidase conjugate Ab (1:20,000), using an ECL system (Thermo Fisher). Blots were reprobed with rabbit β-actin mAb (1:1000) to confirm the visible band. Analysis of antibody staining was performed by densitometry [32].

### Statistical analysis

Results were expressed as mean ± standard error mean (SEM). Differences of diet on maternal and fetal outcomes were assessed using a non-parametric Mann-Whitney U test. Statistical significance was set at *p* < 0.05, and data analysis was performed with the statistical Package for Social Sciences (SPSS version 22.0, Chicago, IL, USA).

## Results

### Dietary free or bound fructose effect maternal feed and drink intake, and body weight changes of dams and pups

The rats were administered either plain water (control) or water with the polysaccharide maltodextrin (vehicle), or the same amount of added sugar types i.e. sucrose, fructose, and HFCS with a concentration of 20% (*w*/*v*), as shown in Table 2. Energy, carbohydrate, protein, fat, fiber, and micronutrient contents were similar in all four diets, except the regular control diet. The control group received feed and plain water (30.6 ± 1.3 kcal/day). Dietary groups received feed and sweetened water with added sugars (20%, w/v) including sucrose, fructose, or HFCS (97.6 ± 1.7, 81.9 ± 4.4, and 108.3 ± 3.8 kcal/day, respectively). As an energy control (vehicle) for added sugar in water, the complex carbohydrate maltodextrin was used (80.2 ± 3.4 kcal/g) (Table 2). Total energy and carbohydrate intake of HFCS, sucrose, and fructose groups were higher than those in the control group (*p* < 0.05). The HFCS and sucrose groups consumed more of the water with added sugar than the maltodextrin group (*p* < 0.05), while there was no significant difference in the water intake of the fructose and maltodextrin groups. Because of the differences in feed intake, the overall fat and protein intake of the HFCS group were the highest, and the sucrose group consumed more fat and protein than the control and maltodextrin groups (*p* < 0.05).

**Table 2:** Daily energy and macronutrient intake of the animals during the dietary manipulation

	Diet groups (M ± SEM)				
	Control	Maltodextrin	Sucrose	Fructose	HFCS
Energy (kcal/day)	48.5 ± 2.0	99.7 ± 4.1	123.0 ± 2.3^*#^	102.9 ± 6.1^*^	135.6 ± 5.4^*#^
Carbohydrate (g/day)	6.9 ± 0.3	19.3 ± 0.8	23.4 ± 0.4^*#^	19.6 ± 1.0^*^	26.0 ± 0.9^*#^
Carbohydrate(kcal/day)	27.7 ± 1.1	77.1 ± 3.3	93.5 ± 1.7^*#^	78.5 ± 4.1^*^	103.9 ± 3.5^*#^
Added sugar (g/day)	–	11.7 ± 0.6	13.5 ± 0.5^*#^	11.5 ± 0.5^*^	15.4 ± 0.4^*#^
Added sugar (kcal/day)	–	46.9 ± 2.5	54.1 ± 1.9^*#^	46.1 ± 1.8^*^	61.7 ± 1.8^*#^
Protein (g/day)	3.2 ± 0.1	3.5 ± 0.2	4.6 ± 0.2^*#^	3.8 ± 0.3	4.9 ± 0.3^*#^
Protein (kcal/day)	12.9 ± 0.5	14.1 ± 0.6	18.4 ± 0.7^*#^	15.2 ± 1.2	19.7 ± 1.2^*#^
Fat (g/day)	0.7 ± 0.0	0.8 ± 0.0	1.0 ± 0.0^*#^	0.8 ± 0.1	1.1 ± 0.1^*#^
Fat (kcal/day)	6.2 ± 0.3	6.8 ± 0.3	8.9 ± 0.3^*#^	7.3 ± 0.6	9.5 ± 0.6^*#^

HFCS: High Fructose Corn Syrup; M: Mean; SEM: Standard Error of Mean

*p < 0.05 compared to control, #*p* < 0.05 compared to maltodextrin

**Fig. 2:**
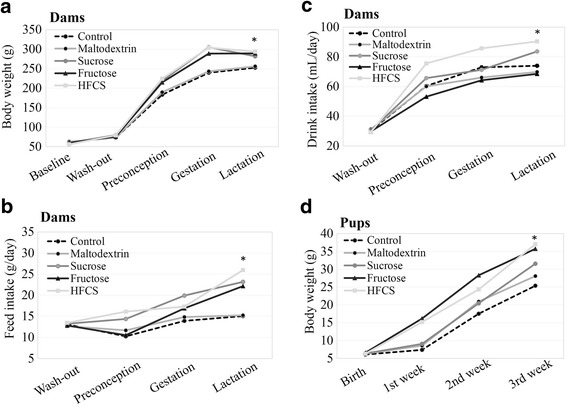
Effect of dietary free or bound fructose on maternal feed and water intake, and body weight changes of dams and pups. **a** Body weights of dams during the study; **b** Daily feed intake of dams during the study; **c** Daily intake of water with added sugar or plain water of dams during the study; **d** Birth weights and body weight changes of pups till weaning. Means (nd_ams_ = 5–7 per group; n_pups_ = 6–7 per group). *p < 0.05 vs. vehicle (maltodextrin)

Maternal feed intake, consumption of the water with added sugar, and body weight changes are presented in Fig. 2. Body weight, and feed and water intake of the animals in all of the groups were similar at the baseline measured during the washout period (2 weeks) prior to the 18-week dietary period (Fig. 2a). The mean body weights (60.4 ± 4.6 g) as well as the feed and water intake of the dams at the start of the experiment did not vary significantly (Fig. 2a-c) (*p* > 0.05). During the 18-week dietary period, body weight changes, and feed and water intake were elevated differently in each group (Fig. 2a-d). Weight gain, and feed and water intake differences began preconception and continued through the gestation and lactation periods. Terminal body weights of dams in the HFCS group (293.7 ± 9.9 g) were the highest, followed by the fructose group (289 ± 6.4 g), and the sucrose group (282.0 ± 13.9 g), as compared to the maltodextrin group (258.3 ± 17.4 g) and the control group (252.9 ± 11 g) (Fig. 2a) (*p* < 0.05). In agreement with this, the daily feed intakes were the highest in the HFCS group, and the second highest in the fructose group (Fig. 2b). Although the average daily energy intake in all dietary periods was the highest in the HFCS group, the body weight gain of the fructose group was not significantly less than in the HFCS group (*p* > 0.05).

All animals became pregnant and two rats failed to carry pregnancy to the third week of gestation. The litter size at birth did not vary significantly between the groups, but there was an effect of diet exposure for dams on the overall litter size. Body weights of the pups at baseline were approximately 6.3 ± 0.3 g (*p* < 0.05), and after weaning the body weights of the pups in the HFCS group (37.0 ± 2.2 g) were the highest, followed by the fructose group (35.7 ± 2.4 g), and the sucrose group (31.6 ± 2.4 g), as compared to the maltodextrin (28.1 ± 2.2 g) and control groups (25.4 ± 1.8 g) (Fig. 2d) (*p* < 0.05).

### Dietary free or bound fructose influence maternal and fetal glucose metabolism and insulin resistance

**Fig. 3:**
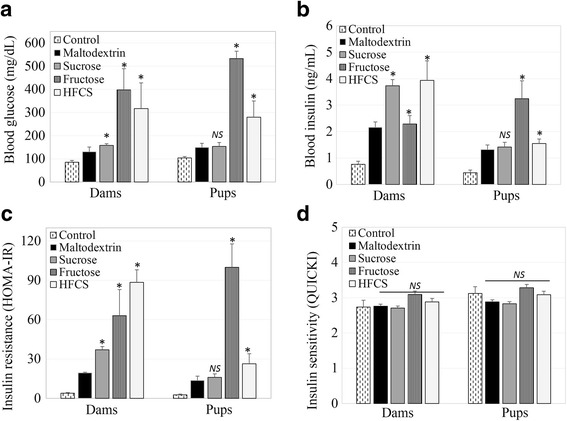
Influence of dietary free or bound fructose on maternal and fetal glucose metabolism and insulin resistance. **a** Blood glucose levels of dams and pups; **b** Blood insulin levels of dams and pups; **c** Blood insulin resistance of dams and pups; **d** Blood insulin sensitivity of dams and pups. Means ± SEM (n_dams_ = 5–7 per group; n_pups_ = 6–7 per group); *p < 0.05 vs. vehicle (maltodextrin); NS, not significant

After the 18-week dietary period, blood plasmas from all rats (dams and pups) were analyzed for fasting glucose and fasting insulin levels (Fig. 3a, b). The blood glucose levels of dams in the fructose (397.5 ± 91.5 mg/dL), HFCS (316.6 ± 111.4 mg/dL), and sucrose (158.1 ± 7.5 mg/dL) groups were higher compared to the maltodextrin (130.4 ± 20.3 mg/dL) and control (85.2 ± 7.7 mg/dL) groups (*p* < 0.05). In parallel, the blood glucose levels of the pups were higher in the fructose (532.79 ± 32.1 mg/dL) and HFCS (279.4 ± 70.1 mg/dL) groups, as compared to the maltodextrin (149.5 ± 17.7 mg/dL) and control (103.6 ± 6.2 mg/dL) groups (*p* < 0.05). However, sucrose (153.9 ± 16.9 mg/dL) intake in pups did not significantly affect the fasting blood glucose level as compared to maltodextrin (*p* > 0.05) (Fig. 3a). The fasting serum insulin levels of dams in the HFCS (3.9 ± 0.7 ng/mL), sucrose (3.74 ± 0.2 ng/mL), and fructose (2.3 ± 0.3 ng/mL) groups were higher compared to the maltodextrin (2.2 ± 0.2 ng/mL) and control (0.8 ± 0.1 ng/mL) groups (p < 0.05). In pups, the blood insulin levels observed in the fructose (3.24 ± 0.7 ng/mL) and HFCS (1.55 ± 0.2 ng/mL) groups were elevated as compared to the maltodextrin (1.33 ± 0.2 ng/mL) and control (0.45 ± 0.1 ng/mL) groups (*p* < 0.05). However, sucrose (1.42 ± 0.2 ng/mL) intake in pups did not significantly affect fasting blood insulin levels, as compared to maltodextrin (*p* > 0.05) (Fig. 3b).

To estimate the effect of dietary free or bound fructose on insulin sensitivity and insulin resistance, HOMA-IR and QUICKI were calculated from fasting blood glucose and insulin levels (Fig. 3c, d). The HOMA-IR of the dams were higher in the HFCS (63.18 ± 36.18), fructose (50.47 ± 21.25), and sucrose (26.39 ± 7.14) groups as compared to the maltodextrin (7.78 ± 4.77) and control (2.36 ± 1.01) groups (Fig. 3c) (*p* < 0.05). The HOMA-IR values of the pups were the highest in the fructose (79.94 ± 31.34) group, followed by the HFCS (15.04 ± 7.58), maltodextrin (8.15 ± 4.10), sucrose (6.82 ± 3.60) and control (1.54 ± 0.82) group (*p* < 0.05). Conversely, the insulin resistance in the sucrose group of pups was not significantly different from the maltodextrin group (*p* > 0.05) (Fig. 3c). Furthermore, the insulin sensitivity scores of the dams estimated by QUICKI were comparable in the fructose (3.09 ± 0.14), HFCS (2.88 ± 0.14), maltodextrin (2.76 ± 0.07), control (2.73 ± 0.08), and sucrose (2.70 ± 0.03) groups (Fig. 3d) (*p* > 0.05). Similarly, the QUICKI values of the pups were comparable in the fructose (3.28 ± 0.03), control (3.12 ± 0.20), HFCS (3.08 ± 0.14), maltodextrin (2.88 ± 0.01), and sucrose (2.83 ± 0.07) groups (Fig. 3d) (*p* > 0.05).

### Dietary free or bound fructose Alter maternal and fetal circulating free fatty acids, triglycerides and fatty acid synthesis

**Fig. 4:**
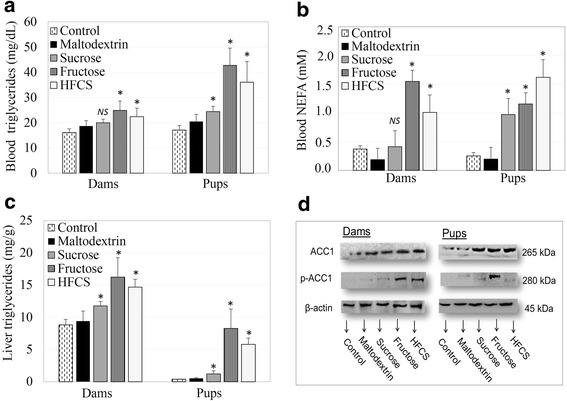
Outcome of dietary free or bound fructose on maternal and fetal circulating free fatty acids, triglycerides, and fatty acid synthesis. **a** Blood triglycerides levels of dams and pups; **b** Blood non-esterified fatty acids (NEFA) levels of dams and pups; **c** Liver triglyceride levels of dams and pups; **d** Acetyl Co-A Carboxylase (ACC1) and phosphorylated forms (p-ACC1) expression estimated by western blot in liver tissue of dams and pups. Means ± SEM (n_dams_ = 5–7 per group; n_pups_ = 6–7 per group); *p < 0.05 vs. vehicle (maltodextrin); NS, not significant

Blood plasma and livers from all of the rats were analyzed for circulating triglycerides, NEFA, liver triglycerides, and a liver fatty acid synthesis rate limiting enzyme (ACC1) after dietary exposure for 20 weeks, including the preconception, gestation, and lactation periods. The effect of dietary free or bound fructose on these biomarkers of de novo lipogenesis are shown in Fig. 4.

Blood triglyceride content was the highest in dams and pups in the fructose group (24.9 ± 3.8; 42.8 ± 6.8 mg/dL), followed by the HFCS group (22.4 ± 3.4; 36.1 ± 8.2 mg/dL) and the sucrose group (20.0 ± 1.4; 24.4 ± 2.1 mg/dL), as compared to the maltodextrin (18.7 ± 2.2; 20.5 ± 2.9 mg/dL) and control groups (16.1 ± 1.5; 17.1 ± 1.7 mg/dL) (*p* < 0.05). However, the blood triglyceride content in dams from the sucrose group was not significantly different from that in the maltodextrin group (*p* > 0.05) (Fig. 4a). Blood NEFA levels were higher in dams and pups of the HFCS (0.9 ± 0.2; 1.1 ± 0.3 mM), fructose (0.7 ± 0.1; 0.9 ± 0.1 mM), and sucrose (0.5 ± 0.1; 0.8 ± 0.1 mM) groups as compared to the maltodextrin (0.4 ± 0.04; 0.6 ± 0.03 mM) and control groups (0.4 ± 0.1; 0.7 ± 0.1 mM) (Fig. 4b) (*p* < 0.05).

The total triglyceride content per gram of liver tissue were the highest in the dams and pups from the fructose group (16.2 ± 5.3; 8.3 ± 1.6 mg/g), followed by the HFCS group (14.7 ± 1.2; 5.8 ± 0.9 mg/g) and the sucrose group (11.8 ± 0.7; 1.2 ± 0.5 mg/g), as compared to the maltodextrin (9.4 ± 1.6; 0.5 ± 0.1 mg/g) and control (8.8 ± 0.9; 0.4 ± 0.1 mg/g) groups (Fig. 4c) (*p* < 0.05). To measure the rate of fatty acid synthesis, the regulator enzyme ACC1 was detected by western blot in livers. Liver samples were analyzed for ACC1 and p-ACC1 in homogenized tissue samples (Fig. 4d). Fructose and HFCS exposure resulted in thicker p-ACC1 bands on western blot membranes as compared to the maltodextrin and control groups, indicating a more active fatty acid synthesis pathway.

## Discussion

Studies showed that fructose taken from natural sources such as honey and fruits had more positive and less negative effects on weight loss, waist hip ratio and hypertriglyceridemia [20, 21] compared to industrial fructose. Honey and fruits contain fructose as well as fiber and antioxidants such as vitamin C, resveratrol and flavonols. Thus, unlike added sugars fruits and honey can be evaluated as healthy fructose sources despite the relatively high fructose content [20, 33–35]. However, added fructose should be considered different from natural fructose. Because many studies reported that; added fructose intake might be related to elevated plasma triglyceride levels [36, 37], hypertension [38], increased body weight [39], increased plasma insulin and glucose levels [37, 40] and hepatic insulin resistance [41]. However, most of these studies are hyper caloric or have high fructose content (most people cannot get that much fructose on a standard daily diet).

Since there is clear evidence that exposure to high dietary sugar during pregnancy is associated with an increased predisposition to obesity, dietary recommendations mostly tend to emphasize reducing added sugars [1, 2]. However, whether all added sugar types (free or bound forms) induce a similar metabolic response on the developmental programming of lipogenesis needs to be clarified. Therefore, this study investigated the effect of chronic maternal consumption of bound or unbound fructose in three different added sugars (fructose, sucrose, and high-fructose corn syrup containing 55% fructose) on lipogenesis-related markers.

### Effect of dietary free or bound fructose on maternal feed and drink intake, and body weight changes of dams and pups

During all periods of this study, the energy intakes of all groups were higher as compared to the vehicle and control groups. The daily feed intake of the HFCS group was the highest, and the fructose and sucrose groups consumed more feed than the vehicle and control groups. As published by others, the taste of HFCS may induce increased water consumption, resulting in higher energy intake [16, 42]. On the other hand, excess free fructose intake may decrease insulin and leptin secretion, which in turn may result in less postprandial ghrelin suppression and stimulate more feed intake, as shown by others [15, 43].

The body weights in the fructose, HFCS, and sucrose groups were higher than in the vehicle and control groups, as indicated by energy intake. In agreement of our results, some studies have shown that fructose intake increases the body weight of rats and mice [11, 44, 45]. Conversely, some studies have reported that HFCS and fructose intake (%10 *w*/*v*) did not affect the body weights of dams [18] or humans [43]. Similar to our results, other studies have reported that maternal fructose (free or in HFCS) intake leads to an increase in the body weight of pups [9, 46, 47]. A study showed that maternal fructose exposure decreased hypothalamic sensitivity to exogenous leptin, enhanced food intake, and decreased several anorexigenic signals [47]. Another study reported that maternal fructose intake lead to alterations in leptin and adiponectin levels [9]. However, further studies are needed to clarify the mechanism underlying the influence of fructose on the developmental programming of obesity. A meta-analysis showed that fructose consumption in isocaloric studies did not change body weight, while hypercaloric studies showed a significant increase [48]. Another meta-analysis reported that 10–20% (w/v) fructose consumption was associated with elevated body weight in rodents [37]. As indicated, some studies have reported an increase in the body weight due to fructose consumption, while other studies showed a decrease, or no change. These controversial results of fructose on body weight might be due to fructose type (bound or free), dose, exposure time, or subjects of the study (human or rodent).

The energy intake through feed and sweetened water of dams in the HFCS group was higher than in the fructose group, but body weight gain was lesser in the HFCS group than in the fructose group in this study. Although fructose (100% free fructose) has almost the same energy content as HFCS-55 (including 55% free fructose) and sucrose (including 50% bound fructose), more energy intake did not result in body weight gain in the HFCS and sucrose groups as compared to the fructose group. This might reveal the effect of free fructose-induced lipogenesis and body weight elevation, as compared to bound fructose (sucrose) [44].

Fructose and HFCS intake affect the body weight of dams in a same way, but in this study only HFCS intake resulted in elevated body weight of pups during the 3 weeks. In parallel with this data, even a 10% (*w*/*v*) fructose-containing maternal diet caused body weight increase in pups, as shown recently by others [9]. These results might be related to the HFCS-induced fetal programming of lipogenesis via hormonal (insulin) or enzymatic (ACC1) pathways [49, 50].

### Influence of dietary free or bound fructose on maternal and fetal glucose metabolism and insulin resistance

To elucidate the hormonal control of the lipogenesis, blood glucose, and insulin content, insulin resistance and insulin sensitivity were analyzed. Blood glucose levels were the highest in the fructose group, following by the HFCS and sucrose groups, as compared to the vehicle and control groups. Thus, maternal fructose consumption resulted in elevated fasting blood glucose of pups in this study. In this regard, a meta-analysis reported that 10–21% (w/v) fructose consumption is associated with elevated blood glucose and insulin levels [37]. Accordingly, one recent paper reported that a high fructose diet in rats adversely affects glucose tolerance and insulin resistance [51]. Thus, fructose intake over a long period might alter the insulin signaling pathways and cause hyperglycemia and hyperinsulinemia, as shown by other studies [52, 53]. Additionally, fructose intake in the maternal diet increased peak glucose, decreased glucose tolerance [9], increased serum insulin levels and altered insulin signal transduction as compared to glucose, as shown by other studies [54]. High glucose intake via in the form of sucrose or HFCS, triggers insulin secretion. However, high intake of free fructose causes an indirect increase of blood insulin. Fructose may cause an increase in blood NEFA level and liver triglycerides, which might disturb insulin receptors and signaling, resulting insulin resistance [55]. The consequences of fructose consumption on fetal developmental programming needs to be studied further.

To estimate insulin resistance, the HOMA-IR and QUICKI indexes were calculated from fasting glucose and fasting insulin levels. The HOMA-IR of the dams was the highest in the HFCS and fructose groups, as compared to the sucrose group in this study. The HOMA-IR of pups was the highest in the fructose and HFCS groups, as compared to the sucrose group. Similarly, Saad et al. reported elevated HOMA-IR scores in a group of pups exposed to maternal fructose intake [9]. Also, in another study, perinatal fructose consumption increased the fasting insulin levels and HOMA-IR scores of pups [56]. In this study, the HOMA-IR value of the maltodextrin group was higher than expected. A previous study found that there were different gastric emptying rates of different carbohydrates, and more water soluble carbohydrates, like maltodextrin, might leave the gastrointestinal tract faster [57, 58], which may explain this result. However, there was no significant difference between the different types of added sugars on the insulin sensitivity of dams and pups. Free fructose caused insulin resistance, but did not affect insulin sensitivity in this study. However, a study showed that fructose-fed animals have a low score in QUICKI as compared to the control group [59]. Thus, a higher value of HOMA-IR and a lower value of QUICKI mean higher levels of insulin or glucose and an increased risk of insulin resistance [60].

HFCS resulted in more insulin resistance in dams, but free fructose had a more robust effect on the developmental programming of insulin resistance in pups. The effect of HFCS may be more due to the free fructose in HFCS than sucrose. This may be proof that maternal intake of added sugar might alter the development of glucose and insulin regulation pathways, β cell function of the fetus, and may cause predisposition to diabetes. In parallel with this hypothesized mechanism, other studies have shown that maternal fructose intake during pregnancy elevated plasma insulin and glucose levels in pups [61, 62]. Maternal hyperglycemia may cause glucose transition via the placenta, and glucose transition might cause excess fetal insulin secretion. Alternatively, maternal hyperinsulinemia might lead to insulin transition to the fetus, resulting in fetal hyperinsulinemia. Maternal blood glucose or insulin-induced fetal hyperinsulinemia might cause hyperplasia or hyperactivity of pancreatic β cells, leading to glucose tolerance abnormalities or insulin resistance [63, 64].

### Outcome of dietary free or bound fructose on maternal and fetal circulating free fatty acids, triglycerides and fatty acid synthesis

To examine the hypothesis that fructose-induced steatosis might disturb insulin receptors and signaling, maternal and fetal NEFA, triglycerides, and the fatty acid synthesis regulating ACC1 enzyme were analyzed. In this study, not only blood, but also liver triglyceride contents were the highest in the fructose group, followed by the HFCS group and the sucrose group, as compared to the maltodextrin and control groups in dams and pups. Many studies have shown that high fructose intake might increase blood and liver triglyceride levels [46, 52, 65], hepatic fatty acid synthesis [11] and ACC1 enzyme expression [11, 52]. In parallel with this data, a study showed that HFCS- and fructose-supplemented water consumption elevated serum triglyceride levels as compared to sucrose-supplemented water [66]. Sanchez Lozada et al. showed that both fructose (30%) and glucose (30%) intake increased blood triglyceride and cholesterol levels compared to sucrose (60%) [67]. Similar results were also shown in humans: Hochuli et al. reported that moderate fructose (40 g/day fructose for 3 weeks) intake induced de-novo fatty acid synthesis compared to high sucrose consisting diet (80 g/day) [68]. Furthermore, in a meta-analysis, 10–21% (*w*/*v*) fructose was associated with elevated blood triglyceride levels [37]. On the other hand, there are some studies reported conflicting results. These studies are mostly different from ours in terms of the study design. For example, compared to our study Toop et al. [18] administered less sugar (10% vs 20%) for a shorter duration (10 weeks vs 21 weeks) to the rats. In a clinical trial, it was found that a three-day consumption of fructose (52 g) lowered insulin levels but did not differ from sucrose intake (65 g) in terms of plasma triglyceride levels [69]. In this study, administered fructose amount and three-day intervention may not suitable enough to see overall effects of added sugars on lipid profile. Thus, the differences in the design of the may cause conflicting results.

Moreover, free fructose and HFCS had a robust effect of increasing blood and liver triglyceride and NEFA contents in pups as compared to dams. This might give a clue for the developmental/fetal programming of lipogenesis. Conversely, in some studies, HFCS intake did not change body weight, triglyceride [18] and apoprotein B [70] levels as compared to sucrose in adults. Moreover, while the metabolism of fructose does not stimulate insulin secretion as much as glucose, high amounts of fructose may contribute to de novo lipogenesis in liver, which is the main regulator organ for fructose metabolism [71]. Not only being free or bound to another molecule, but also the type of molecule (D or L type fructose) might be important in metabolic regulation and integration [72]. However, studies comparing the metabolism by the type (D or L type) or form (bound, free) of fructose are limited.

We observed that blood NEFA content elevated after HFCS intake in both dams and pups. Correspondingly, some studies have reported that high fructose intake increases NEFA and causes dyslipidemia and hepatic lipid accumulation [73, 74]. Nevertheless, maternal high fructose intake did not increase the blood NEFA levels of dams, as compared to glucose, shown by other studies [54]. Liu et al. reported that high fructose intake increases both plasma NEFA levels and oxidation, resulting in stimulation of excess insulin secretion and insulin resistance [75]. Additionally, it was shown that acylation stimulating protein (ASP) might stimulate NEFA oxidation and lipolysis in adipocytes [76]. Therefore, high fructose intake might increase de novo lipogenesis and fatty acid acylation; resulting in high plasma triglyceride and NEFA levels.

ACC1 plays an essential role in the regulation of fatty acid synthesis and degradation. This enzyme catalyzes the rate-limiting step in fatty acid synthesis at the level of production of malonyl-CoA. Some studies have shown that high fructose intake might lead to relatively higher ACC1 and pACC1 expression [77, 78]. Accordingly, other studies showed that fructose may activate transcription factors such as sterol regulatory element-binding proteins (SREBP-1c and ChREBP), which control de novo lipogenesis enzymes such as ACC1 and fatty acid synthase (FAS) [14, 52, 79]. In support of this mechanism, we presented that a higher level of the activated enzyme p-ACC1 caused more fatty acid synthesis in the fructose and HFCS groups.

## Conclusions

In this study, we showed an adverse effect of free fructose on body weight, plasma glucose, insulin responses, and liver lipogenesis parameters, as compared to bound fructose, in both dams and their pups. Additionally, our study found that fructose intake with maternal diet might affect insulin resistance and lipogenesis in the adult life of the pups. This might be evidence for the role of free fructose on developmental programming. However, free fructose in natural foods itself could not cause negative health effects in small amounts taken via healthy diet. Fructose dose and exposure time may be the reason of conflicting results between studies. To explain the exact mechanisms of metabolic changes, adipose deposition, adipokines, insulin signal transduction, and transcriptional factors must be evaluated in future studies. In conclusion, bound or free high fructose intake may increase the risk for chronic diseases, especially obesity and type II diabetes mellitus. Thus, free fructose, as that found in HFCS, contributes to chronic disease development more than bound fructose does, similar to sucrose. Different types of added sugar in the maternal diet result in different metabolic responses in the developmental programming of lipogenesis.

## Abbreviations

Ab: Antibody
ACC: Acetyl Co-A carboxylase
ASP: Acylation stimulating protein
ChREBP: Carbohydrate-responsive element-binding protein
CVD: Cardiovascular diseases
ECL: Enhanced chemiluminescence
FAS: Fatty acid synthase
HFCS: High fructose corn syrup
HOMA-IR: Homeostasis model assessment of insulin resistance
HRP: Horseradish peroxidase
mAb: Monoclonal antibody
NEFA: Non-esterified fatty acids
NP-40: Nonidet P-40
QUICKI: Quantitative insulin sensitivity check index
SDS-PAGE: Sodium dodecyl sulfate polyacrylamide gel electrophoresis
SEM: Standard error of mean
SREBP-1c: Sterol regulatory element binding protein-1c
T2DM: Type 2 diabetes mellitus
